# Helium structures around SF_5_^+^ and SF_6_^+^: novel intermolecular potential and mass spectrometry experiments†

**DOI:** 10.1039/d1cp04725f

**Published:** 2021-12-17

**Authors:** Eva Zunzunegui-Bru, Elisabeth Gruber, Stefan Bergmeister, Miriam Meyer, Fabio Zappa, Massimiliano Bartolomei, Fernando Pirani, Pablo Villarreal, Tomás González-Lezana, Paul Scheier

**Affiliations:** Instituto de Física Fundamental, IFF-CSIC Serrano 123 28006 Madrid Spain t.gonzalez.lezana@csic.es; Universität Innsbruck, Institut für Ionenphysik und Angewandte Physik Technikerstraße 25 6020 Innsbruck Austria; Dipartimento di Chimica, Biologia e Biotecnologie, Universitá di Perugia 06123 Perugia Italy

## Abstract

Helium clusters around the recently experimentally observed sulphur hexafluoride SF_6_^+^ and sulphur pentafluoride SF_5_^+^ ions are investigated using a combined experimental and theoretical effort. Mass spectrometry ion yields are obtained and the energetics and structure of the corresponding He_*N*_–SF_6_^+^ and He_*N*_–SF_5_^+^ clusters are analyzed using path integral molecular dynamics calculations as a function of *N*, the number of He atoms, employing a new intermolecular potential describing the interaction between the dopant and the surrounding helium. The new force field is optimized on benchmark potential energy *ab initio* calculations and represented by improved Lennard-Jonnes analytical expressions. This procedure improves the previous potentials employed in similar simulations for neutral SF_6_ attached to helium nanodroplets. The theoretical analysis explains the characteristic features observed in the experimental ion yields which suggest the existence of stable configurations at specific sizes.

## Abstract 2

The structure of the He atoms around SF_5_^+^ and SF_6_^+^ is investigated both experimentally and theoretically.

## Graphical abstract

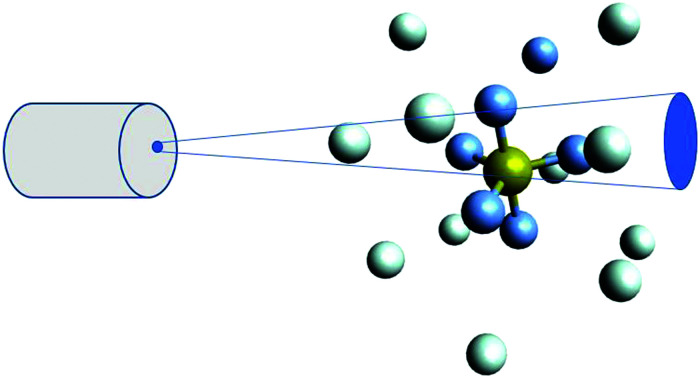

## 1 Introduction

Sulphur hexafluoride SF_6_ embedded in an environment formed by helium nanodroplets (HNDs) was widely investigated by different groups in the late 1990s. The pioneering vibrational spectroscopy investigation carried out by Goyal *et al.*,^1,2^ using SF_6_ as the dopant molecule, was followed by intensive high resolution infrared spectroscopy by Toennies, Vilesov and collaborators.^3–6^ In particular, in ref. 4 rotational constants and temperature were obtained from the first report of a rotationally fully resolved infrared spectrum of the SF_6_ molecule embedded in liquid helium. A droplet temperature of 0.37 K was considered cold enough to display superfluid behaviour and the rotational constants associated with the He_*N*_SF_6_ droplet were found to be consistent with about eight He atoms rigidly attached to the molecular frame.^4^ These doped helium clusters were then the subject of a large number of theoretical calculations, usually employing Monte Carlo methods, focused on understanding both the structure of the helium layers surrounding the impurity and its rotation within the superfluid droplet.^7–14^ Since the main goal of most of those previous studies was to investigate whether or not the solvating helium displays superfluid behavior, the focus of the analysis usually was on superfluid fractions and radial probability densities, but little was said about the precise structure of the He atoms around the impurity for small clusters.

In contrast to all this vast bibliography on sulphur hexafluoride, its ionic counterpart, SF_6_^+^, has received considerably less attention. One of the reasons for this is its elusive character which has prevented a reliable experimental detection to date. After a long list of unsuccessful attempts at both observing SF_6_^+^ ^15–22^ and stabilizing this ionic species in SF_6_ clusters,^23–25^ the search for the transient sulphur hexafluoride cation ended with the report of its stabilization using helium nanodroplets (HNDs).^26^ Albertini *et al.* demonstrated that sufficiently long-lived SF_6_^+^ can be formed by doping charged helium nanodroplets with neutral SF_6_.^26^ The ions are identified by means of high-resolution mass spectrometry and collision-induced dissociation following the collision of helium gas with mass-selected He_*N*_SF_6_^+^.

Although it seems well accepted that the extra F′ atom of SF_6_^+^ (as compared to SF_5_^+^) is assumed to occupy an external position forming a weakly bonded complex F′–SF_5_^+^,^27–30^ not too much information regarding the structure of He atoms around the cation can be found in the literature. In their study, Albertini *et al.* had not paid much attention to the position of the He atoms with respect to SF_6_^+^ due to the minor importance of the energetic considerations in elucidating the precise decomposition channels followed in order to produce either the sulphur hexafluoride or pentafluoride cations.^26^ The authors concede, nevertheless, that the formation of helium snowball cage structures surrounding SF_5_^+^ may play a significant role in delaying the recombination of the pair SF_5_^+^ + F.^26^ It is therefore of interest to analyze the pattern followed by He atoms during their solvation of the dopant. In this work, we analyze in detail the clusters formed by He atoms around both cations, SF_5_^+^ and SF_6_^+^, using path integral molecular dynamics (PIMD) calculations.

For this kind of investigation, the potential describing the interaction between the dopant and the surrounding helium has to be as accurate as possible. However in most of the previous theoretical studies on neutral SF_6_ attached to HNDs, the intermolecular potential was described using simple empirical two-body (2B) expressions with coefficients and parameters refined by simultaneous fittings of properties such as differential cross-sections, viscosities and virial coefficients.^31–34^ Some of them were just simple spherical 12-6 or exp-6 potentials with dependence on the experiment considered for the fitting. Typical issues considered in these investigations on the neutral sulphur hexafluoride attached to rare gas droplets are the possible spherical character of octahedral molecules and the manifestation of anisotropic effects.^31,35–37^ Here, for this work, we have developed a new potential energy surface (PES) to describe the existing molecular interactions. As in previous studies on doped HNDs^38–40^ benchmark *ab initio* calculations have been carried out and the resulting potential energy points are optimised and represented *via* an improved Lennard-Jones (ILJ) expression.

The structure of the paper is as follows. in Section 2 we present the experimental work and in Section 3 we discuss the theoretical part of our study. In particular, Section 3.1 is devoted to the calculation of the intermolecular potential employed in our simulations and in Section 3.3, brief details of the PIMD method are given. Results are shown in Section 4 and conclusions are drawn in Section 5.

## 2 Experiment

**Fig. 1:**
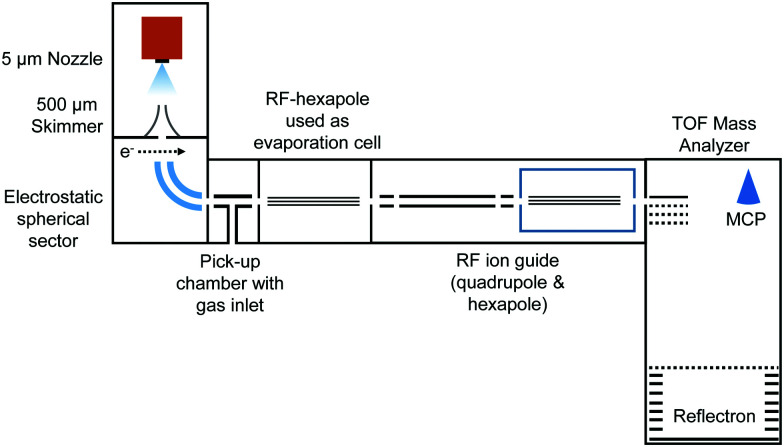
Schematic representation of the experimental setup.

In the present work, charged HNDs were doped with SF_6_ in a pick-up chamber and then gently shrunk in a separate evaporation chamber filled with He gas, thereby leading to the complexation of the dopant ions with a small number of helium atoms accessible for mass spectrometry (see Fig. 1). HNDs were formed by supersonic expansion of pre-cooled and pressurized He gas (28 bar, 99.9999% purity) in vacuum through a 9.8 K cooled 5 μm nozzle. The expanding beam was skimmed by a conical skimmer and then crossed with a beam of electrons (at 38 eV and an emission current of 473 μA). The collision with electrons might lead to the formation of highly charged HNDs,^41^ which were then size-to-charge (*N*/*z*) selected by passing a 90° electrostatic spherical sector. The presented measurements were performed by tuning the sector to *N*/*z* = 8.8 × 10^4^. After this, the *N*/*z* selected HNDs were guided into a pick-up chamber filled with SF_6_ (0.04 mPa, the measured pressure was corrected by taking the gas correction factor of SF_6_ into account) at room temperature. Collisions between the charged HNDs and SF_6_ led to doping of the droplets and to the formation of SF_6_^+^ (SF_5_^+^F, see ref. 26) and [(SF_6_)_*n*_SF_5_]^+^ clusters embedded in the HNDs. In the following so-called evaporation chamber, the doped HNDs were guided by a RF-hexapole, which was filled with He gas at various pressures (*P*_evap_). Here, collisions between the doped HNDs and He atoms led to a gentle stripping of the helium matrix until bare charged dopants (SF_6_^+^ and SF_5_^+^) and ions complexed with a small number of helium atoms emerged. These ions were then guided by a quadrupole and a hexapole into a commercial time-of-flight mass spectrometer (Micromass Q-TOF Ultima Waters) with a mass resolution of 8000 in the V-mode and 15 000 in the W-mode. The mass spectra were evaluated using the custom-designed software IsotopeFit,^42^ which takes the isotope pattern of the contributing ions into account.

## 3 Theory

### 3.1 Intermolecular potential

In order to precisely predict the structural and energetic features of the He*N*–SF_5_^+^ and He–SF_6_^+^ clusters, the involved intermolecular interaction must be accurately obtained and made available in a suitable analytical form. The force field employed here is indeed based on the sum of 2B He–SF_5_^+^, F′–SF_5_^+^, He–F′ (F′ being the external non-covalently bound F atom) and He–He non-covalent interaction contributions. For the He–He interaction we have used the potential reported in ref. 43 while for the remaining contributions we have developed new PESs.

The global interaction between the SF_5_^+^ ion and an external atom (either F′ or He) can be formulated as a combination of three “effective” components:

1 *V*_inter_ = *V*_vdW_ + *V*_ind_ + *V*_elect_,

which represent the van der Waals (size repulsion plus dispersion attraction), induction and the electrostatic interaction contributions, respectively.

The van der Waals *V*_vdW_ term is expressed as a sum of effective atoms (on SF_5_^+^)-external atom pair-wise contributions2
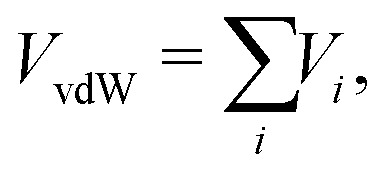
where the sum runs over all possible atoms on the SF_5_^+^ molecule, with each one considered as an effective atom, as they behave differently with respect to the isolated atomic counterparts.

The formulation adopted for each term *V*_*i*_ term in eqn (2) is of the ILJ type:^44^3
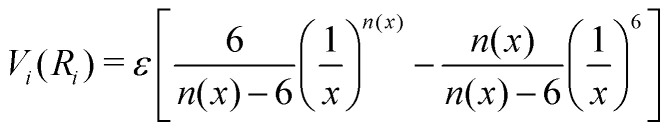
where *x* is the reduced distance of the two bodies defined as4
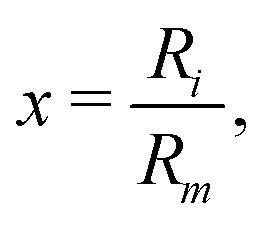
with *R*_*i*_ being the atom–atom distance between an atom on SF_5_^+^ and the interacting partner (either F or He) while *ε* and *R*_*m*_ are respectively the well depth and its position of the interaction potential at the equilibrium value of *R*_*i*_.

The key feature of the ILJ functional form is the adoption of additional (variable) *n* exponential parameters providing more flexibility than the usual Lennard-Jones (12,6) (LJ) ones, thanks to its dependence on *R*_*i*_ as follows:^44^

5 *n*(*x*) = *β* + 4.0 *x*^2^

in which *β* is a parameter depending on the nature and the hardness of the interacting particles leading to a more realistic representation of both repulsion (first term in square brackets of eqn (3)) and attraction (second term in square brackets of eqn (3)).

As for the *V*_ind_ term of eqn (1), it has been introduced to describe the attractive charge-induced dipole contribution determined by the integer positive charge on SF_5_^+^. As suggested by CM5^45^ atomic charge calculations performed at the Hartree–Fock/aug-cc-VTZ^46^ and B3LYP/cc-VTZ^46^ levels of theory, here it is assumed that the whole charge is exclusively borne by the S atom and the used expression is6
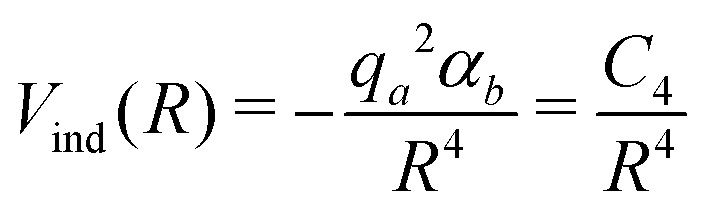
where *q*_*a*_ and *α*_*b*_ are the positive charge on SF_5_^+^ and the static dipole polarizability of the external F′(He) atom, respectively, and *R* is the F′–S (or He–S) distance.

In the study reported here, the *V*_elect_ term of eqn (1) has been introduced only in the case of the SF_5_^+^–F′ interaction by retaining the main charge-quadrupole contribution. In particular, the use of the expression7
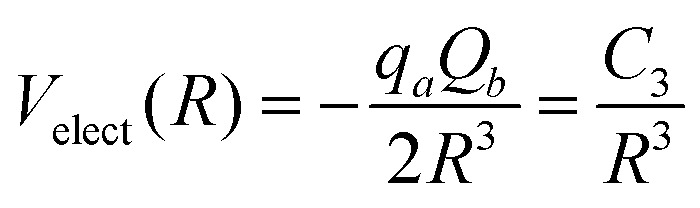
has been made where *q*_*a*_ and *Q*_*b*_ are the positive charge borne by the S atom and the permanent quadrupole moment of the external F′ atom, respectively, and *R* is the F′–S distance. Please note that in the above expression we are considering only the most favorable orientation (90° with respect to *R*, intermolecular distance vector) of the semioccupied orbital of the F′ atom, that is, the orientation leading to the most attractive interaction.

In the case of the He–F′ interaction just the first term in eqn (1) is retained since contributions other than the van der Waals one are not necessary and its representation only involves the formula in eqn (3).

**Fig. 2:**
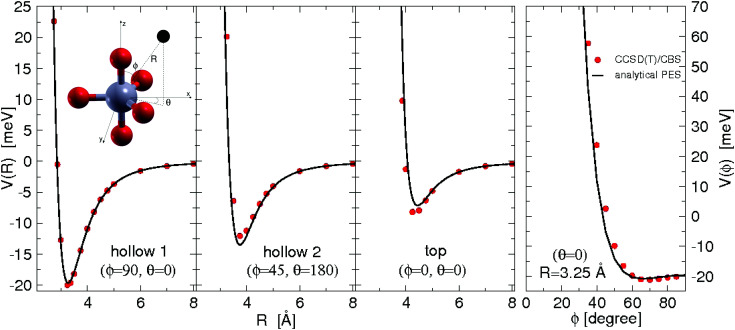
He–SF_5_^+^ intermolecular interaction as obtained from *ab initio* calculations and its analytical representation following eqn (1)–(3). Cuts of the potential (measured in meV) at different values of the angles *ϕ* and *θ* as a function of the distance *R* (in Å) of the He atom with respect to the center of mass of the dopant (see the graphical scheme included in the first panel with the corresponding coordinates). From left to right panels three different positions: (i) hollow 1 with *ϕ* = 90°, *θ* = 0°; (ii) hollow 2 with *ϕ* = 45°, *θ* = 180° and (iii) top with *ϕ* = *θ* = 0°. In the fourth panel, we show the *ϕ* dependence for *R* = 3.25 Å (roughly corresponding to the minimum *R* value for the hollow 1 approach) and, that is, the pathway connecting the hollow 1 and top configurations.

**Fig. 3:**
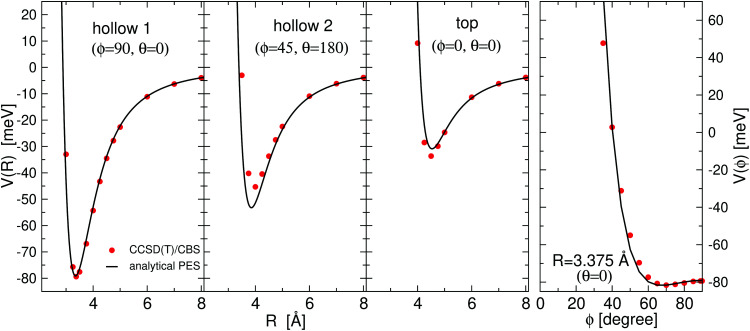
Same as Fig. 2 for the F–SF _5_^+^ interaction.

**Fig. 4:**
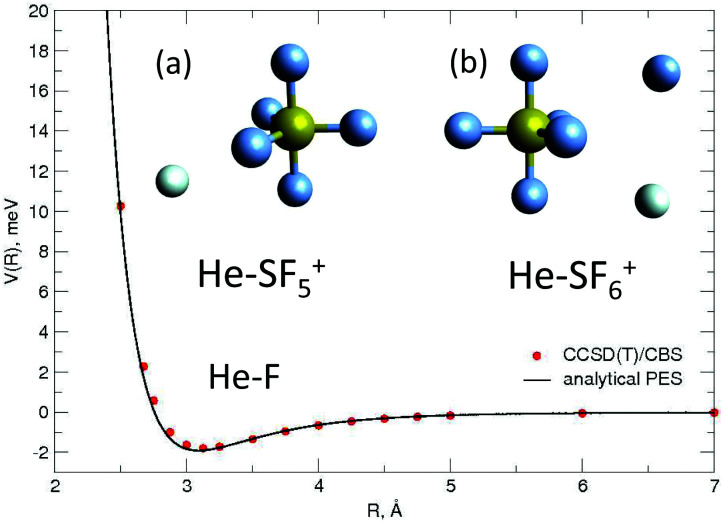
He–F′ interaction potential energy as a function of *R*, the distance between the He and the external F atom. *Ab initio* CCSD(T)/CBS points (red circles) are compared with the ILJ analytical representation (black line). Insets correspond to predictions obtained with the EA for the minimum energy structures for (a) the He–SF_5_^+^ and the (b) the He–SF_6_^+^ systems.

All the used parameters are reported in Table 1. Fine tuning has been carried out for *ε* and *R*_*m*_ by exploiting a comparison with *ab initio* calculations, performed by using the Molpro2012.1 package,^47^ of the intermolecular interaction energies. In particular, the optimisation of the force field has been performed by varying the potential parameters within restricted ranges in order to maintain their correct relation with basic properties of involved partners. This guarantees the correctness of the force field represented in the full space of the relative configurations of the interacting partners. For instance, the parameters of the He–F′ pair fall in the right scale of the experimental determination,^48^ while those of the other pairs scale according to the variation of the electronic polarizability of the interacting partners.^49^ Results obtained for selected configurations of the interacting partners are shown in Fig. 2–4 for the He–SF_5_^+^, F′–SF_5_^+^ and He–F′ interactions, respectively. Benchmark theoretical values for the counterpoise corrected interaction energies have been reported and they have been obtained at the CCSD(T) level of theory with two different basis sets (aug-cc-pVQZ and aug-cc-pV5Z^46^) which allowed the estimation of reliable complete basis set (CBS) extrapolations.^50,51^ In the calculations involving the SF_5_^+^ molecule, the latter has been considered as a rigid body and the used equilibrium geometry is that obtained and reported in the ESI of ref. 26.

Parameters of the force field describing the interaction in the He–SF_5_^+^ and He-(SF_5_F′)^+^ complexes (see eqn (4)–(7))

**Table:** 

F′–SF_5_^+^	*ε* (meV)	*R*_*m*_ (Å)	*β*	*C*_4_ (meV Å^4^)	*C*_3_ (meV Å^3^)
F′–S	4.690	3.676	8	−4032	−2016.2
F′–F	4.540	3.272	8

**Table:** 

He–SF_5_^+^	*ε*	*R*_*m*_	*β*	*C*_4_
He–S	1.914	3.556	8	−1440
He–F	2.240	3.050	8

**Table:** 

He–F′	*ε*	*R*_*m*_	*β*
He–F′	1.909	3.091	9

In general, in Fig. 2–4 a quite good agreement can be observed between the CCSD(T)/CBS *ab initio* results and the analytical representation of the PESs. More in detail, from Fig. 2 and 3 it can be appreciated that the He-SF_5_^+^ and F′-SF_5_^+^ interactions show similar features with potential curves providing minima at close intermolecular distances (differences are around 0.1 Å) but with a global interaction being about four times more attractive in the case of the F′ external partner. Moreover, from the last panel of Fig. 2 and 3 it is evident that the most attractive configurations are those with an external atom around the equatorial region (*ϕ* ∼ 90°) of the SF_5_^+^ molecule while locations of the F close to the polar region (*ϕ* ∼ 0°) provide the least favorable approaches. In fact, the global minimum is found for *θ* = 0° and *ϕ* around 65–70 degrees for both He–SF_5_^+^ and F′–SF_5_^+^ dimers at very close intermolecular distances of around 3.3 Å. Therefore, these results confirm that the F′–SF_5_^+^ interaction is of a non-covalent type, even if it is globally quite strong compared to the remaining He–SF_5_^+^, He–F′ and He–He contributions.

The present two-body model for the representation of the He_*N*_–SF_5_^+^ and He_*N*_–SF_6_^+^ interactions can be considered as appropriate, as shown in the ESI† where the negligible role played by three-body effects is analyzed using *ab initio* calculations. In particular, Fig. S1 of the ESI† reveals a good agreement between the total three-body interaction and that from a pairwise two-body approach as a function of the rotation angle of one He atom with respect to the other one in a He–S–He configuration.

### 3.2 Evolutionary algorithm

The present evolutionary algorithm (EA)^52^ has already been employed before in similar studies of doped helium clusters.^53^ The theoretical foundations of the method can be found in the reference, so here we will refer only to the most relevant aspects. In essence, the algorithm is based on a natural selection procedure consisting of the confrontation between *M* (30) initial populations (parents) and offspring populations obtained from mutations induced in the original ones. The conformational space of the system is explored through the optimization of a fitness function, always searching for the overall minimum energy. Groups of 10 individuals are confronted and the best fit is chosen taking into account a selected energy threshold (10^−4^ meV in our case).

More specifically, initial populations of *M* individuals or clusters consisting of *N* He atoms surrounding either the SF_5_^+^ or the SF_6_^+^ core are generated. Each individual *i* is characterized by the pair of vectors (*x̂*_*i*_, *η̂*_*i*_) representing the 3*N* Cartesian coordinates of the atoms and standard deviations for Gaussian mutations, respectively. Initial values of *η*_*i*_ = 1 and random choices for the positions within a specific range (0, *Δ*), are considered. Thus, each parent creates a single offspring 
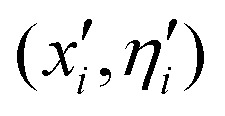
 according to8
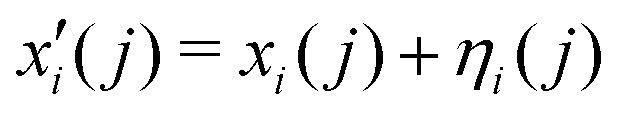
9
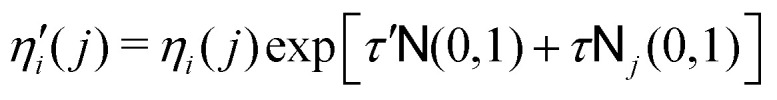
where *j* = 1,…,3*N*; *τ* and *τ*′ are adjustable parameters depending on the value of *N*; 𝒩(0, 1) is a random number from a Gaussian distribution of mean *μ* = 0 and standard deviation *σ* = 1, and 𝒩_*j*_(0, 1) stands for a randomly generated number for each component *j*.

Pairwise comparisons of the energy of each individual with *q* random choices as opponents over the union of 2ℳ elements formed of parents (*x*_*i*_, *η*_*i*_) and offsprings 
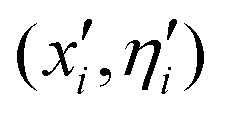
 are performed. Individuals with the lowest energy in their competitions with some other opponents are awarded with points, and finally, those ℳ individuals out of the union of parents and offsprings with a larger number of winning points are selected as survivors to the next generation, thus becoming new parents. The procedure is repeated until the difference between the potential energies of consecutive generations is lower than the above mentioned tolerance value.

### 3.3 Path integral molecular dynamics

When used to calculate static equilibrium properties in the *NVT* ensemble, PIMD provides an approximate description of the effect of quantum fluctuations of the nuclei for a given potential energy model. The path integral calculation can be thermostatted through several schemes as described in ref. 54. In turn, the ring polymer molecular dynamics (RPMD)^55^ approach to get approximate quantum dynamics is defined as evolving under the same choice of Hamiltonian as for the PIMD implementation but with no thermostats turned on. The dynamics generated from these *NVE* trajectories will now be RPMD dynamics. However, due to the ergodicity problems associated with the path integral Hamiltonian one must launch trajectories from many different choices of the initial momenta *i.e.* do a *NVT* thermostatted PIMD run and then launch lots of *NVE* RPMD trajectories from the configurations generated. We restrict ourselves in this work to the first issue, *i.e.* to thermostatted *NVT* simulations, using the i-PI open code of Ceriotti *et al.*^56^

Instead of performing on-the-fly *ab initio* calculations, a capability included in i-PI, and for saving run time, we used the analytic potential model described above. In this model, the SF_5_^+^ core is considered as rigid with the particles arranged at the equilibrium geometry. In order to simulate this behavior in the PIMD runs, the interaction of each pair of particles within the core is described using a very stiff harmonic oscillator with a force constant of 0.01 a.u. This has no consequences for the classical simulation (number of beads *M* = 1), as the system looks for the minimum of the full PES but needs a separate SF_5_^+^ calculation for *M* > 1 (calibration) to be performed. In fact, for *M* = 20 (see below), this rigid compound presents a bond energy of 39.03 meV which has to be subtracted from the energies of different He_*N*_-SF_*n*_^+^ complexes.

Based on the white noise Langevin thermostat, we use the global version of the path integral Langevin equation (PILE-G) stochastic thermostatting scheme^54^ with a unique input parameter *τ*_0_, the friction coefficient which determines the strength of the thermostat. For a temperature of 2 K a value of *τ*_0_= 1 fs was considered along the simulations. When using a large simulation cubic cell (side = 95 Å) it is not necessary to incorporate barostats as the pressure always remains close to zero. A time interval of Δ*t* = 0.1 fs was chosen to be of the order of 1/500 times, the smallest period in the physical system (∼50 fs, corresponding to the maximum kinetic energy of the SF_5_^+^–F′ interaction ∼85 meV), and the quality of the simulation was controlled through the so-called effective energy^57^ in addition to temperature. The latter oscillates around 2 K within 0.05 K while the former is kept within a variation of ∼0.1%.

We carry out the simulations using optimized minimum energy structures obtained by means of the EA^52^ as the initial configuration. Details about this method have been given before and, for an example, we invite the interested reader to examine previous applications.^53^ Thus, starting from those classically estimated minima, we perform the PIMD calculations, first considering a number of beads *M* in the extended system (ring polymer) *M* = 1 (classical), and then *M* = 20. The latter was adopted after using a simple effective atom–atom model for He-SF_5_^+^ which leads, by solving the Schrödinger equation, to a binding energy of 14.89 meV (the PIMD value, at *M* = 20, is 15.19 meV) in such a way that this modest number of beads is able to account for quantum effects, excluding those relative to He–He interactions which would need the use of a huge number of beads (*M* ≥ 500).

Initial velocities, starting from the initial configurations for the complex produced by EA, were generated from a Maxwell–Boltzmann distribution at a given temperature. All the magnitudes were estimated in the centroid approximation.

## 4 Results

### 4.1 SF_5_^+^

**Fig. 5:**
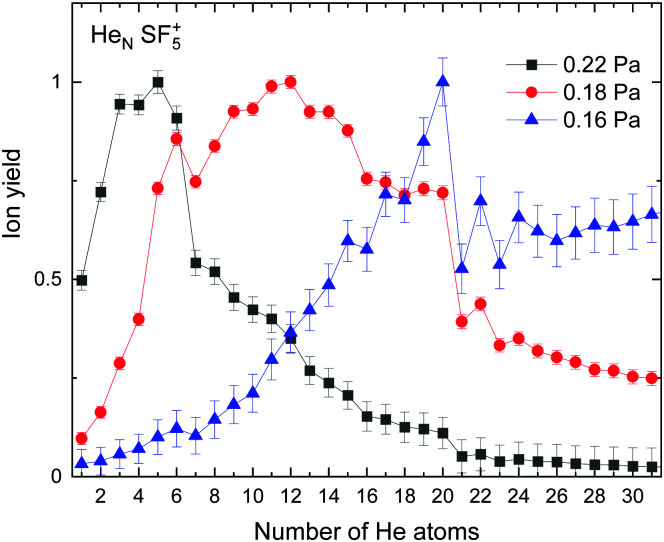
Experimental ion yield for He_*N*_*SF*_5_^+^ for values at gas pressures of 0.16 Pa (blue triangles), 0.18 Pa (red circles) and 0.22 Pa (black squares).

The experimental ion yield observed for He_*N*_–SF_5_^+^ is shown in Fig. 5 for different values of the collision gas pressure employed to remove solvated He from the corresponding ion in order to have a better indication of the existence of possible magic number configurations. Although results certainly differ depending on the gas pressure, specific features are noticeable in the figure for *N* = 6, 12 (in the case of the curve for 0.18 Pa, designated with red circles), 20 and possibly 22 and 24.

Minimum energy structures with a He atom with respect to SF_5_^+^ according to the present force field global optimization process have been obtained using the EA described in Section 3.2. The optimized configuration for He-SF_5_^+^ is shown in panel (a) of the inset of Fig. 4. The classical result reveals the preference of a He atom to occupy a position with values of the spherical angles (see the inset of Fig. 2) of *ϕ* ∼ 66.5° and *θ* ∼ 0° and separated at a distance of about *R* = 3.40 Å from the S atom and about 3.02 Å with respect to the two closest F atoms. There exist, in fact, six symmetrically equivalent minima for either the He atom or the extra F′ atom located at (*θ* = 0°, *ϕ* = 66.5°/113.5°), (*θ* = 120°, *ϕ* = 66.5°/113.5°), and (*θ* = 240°, *ϕ* = 66.5°/113.5°), respectively. We find that this location is also the most favorable site for the extra F′ atom in the sulphur hexafluoride ion.

**Fig. 6:**
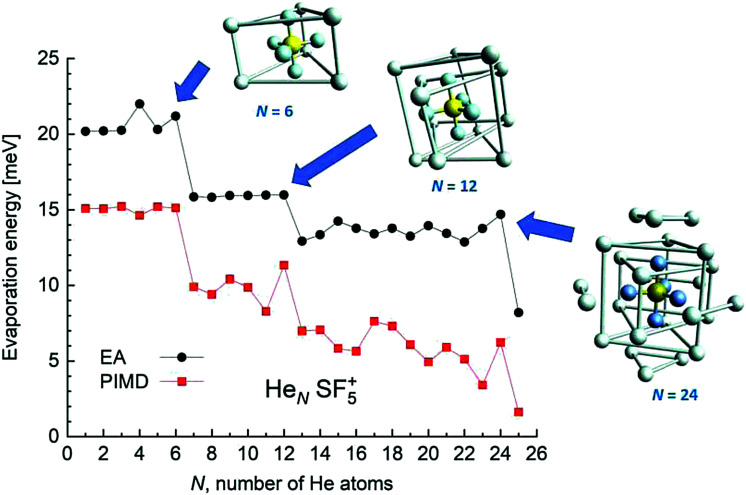
Evaporation energies Δ*E*_*N*_ for the He_*N*_SF_5_^+^ clusters calculated using the EA (black circles) and PIMD (red squares) methods as a function of the number of He atoms, *N*. The figure includes the minimum energy structures observed using the classical EA approach for *N* = 6, 12 and 24 cases. Bonds between some He atoms have been artificially added in order to illustrate the successive helium structures around the dopant.

EA minimization simulations where He atoms are free to move, whereas S and F atoms remain fixed, are carried out to explore the corresponding minimum energy structures for the rest of the He_*N*_*SF*_5_^+^ clusters. Results of the evaporation energies, Δ*E*_*N*_ = *E*_*N*+1_ − *E*_*N*_, as a function of the number of He atoms existing in the clusters are shown in Fig. 6. Three noticeable features are seen at specific sizes. In particular, for *N* = 6, 12 and 24, the Δ*E*_*N*_ curve displays a sudden decrease with respect to the almost average value exhibited for immediately smaller clusters. As in previous investigations in doped helium droplets^38–40^ these effects usually correspond to the filling of a layer or specific caging structures surrounding the dopant. In this case, the analysis of the associated structures for He_6_SF_5_^+^, He_12_SF_5_^+^ and He_24_SF_5_^+^ certainly reveals special arrangements of the He atoms. In particular, for *N* = 6, all equivalent minimum positions shown in Fig. 4 are occupied. This explains that adding an extra helium atom leads to a decrease of the evaporation energy from ∼20 meV to ∼15 meV as observed in Fig. 6, where an inset of the corresponding He_6_SF_5_^+^ structure is included. Analogously, the addition of six more He atoms yields the construction of an outer cage in which extra helium atoms are located in two triangles with vertices facing the vacant F–Ŝ–F angles. For *N* = 24, the classical prediction for the minimum energy geometry seems to correspond to a structure formed with 12 He atoms surrounding the above mentioned He_12_SF_5_^+^ as an internal core. The overall appearance could be understood as triangles, both at the top and bottom, and three pairs of He atoms perpendicular to the planar F atoms.

Fig. 6 also confirms that the QM PIMD calculation seems to confirm the presence of the structures predicted by the EA. Despite its more diffuse trend in comparison with the well-defined plateau regions of the classical result, the quantum evaporation energies shown in Fig. 6 show a qualitatively sudden drop for the same sizes.

**Fig. 7:**
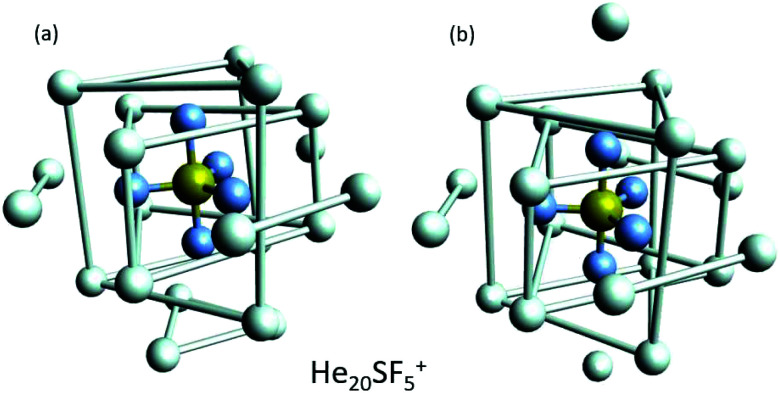
Selected geometries corresponding to He_20_SF_5_^+^. (a) Minimum energy configuration as predicted by EA optimizations and (b) a closed symmetric structure of about 7 meV above the minimum shown in panel (a). As in Fig. 6, He atoms have been artificially bonded as a guide to the eye to distinguish structures around the dopant.

This apparent success of our present calculations to provide some insights regarding the origin of most of the features observed in the experimental ion yields in Fig. 6, contrasts, nevertheless, with the situation for *N* = 20, which remains as an intriguing case, since no clear indications of a specific behaviour with respect to consecutive sizes are seen. The analysis of the minimum energy configuration as predicted by EA optimization, shown in Fig. 7, reveals that He atoms keep the minimum configuration observed for *N* = 12 as an internal core, with the remaining atoms associated with a triangle over the axial straight F–S–F direction, two pairs and an isolated He atom. A perhaps more symmetric closed geometry for such a size of the doped helium cluster is also included in panel (b) of Fig. 7. The *N* = 12-structure is now surrounded by three pairs of He atoms at the plane formed by the S atom and the three central F atoms and two independent atoms both at the two extremes of the F–S–F axis. Its energy is, however, about 7 meV above the minimum energy geometry shown in panel (a).

**Fig. 8:**
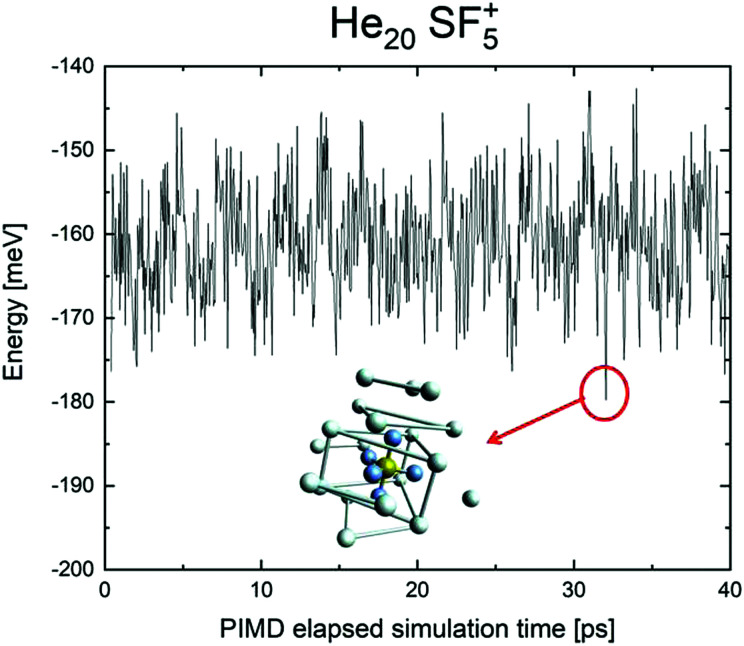
PIMD energy in meV for He_20_SF_5_^+^ as a function of the simulation step. A representation of the geometric centers of the *M* = 20 replica per each He atom yielding the minimum energy during the calculation is also included.

We have tried to grasp a closer insight by analysing the geometrical structure of He_20_-SF_5_^+^. Fig. 8 shows the energies of this droplet as a function of the PIMD simulation step. We have found that helium atoms form configurations during the simulation which are, in essence, small distortions with respect to precisely the minimum energy configurations obtained with EA optimization. In particular, in Fig. 8, we include the structure obtained with the geometrical centers of the *M* = 20 beads employed in the calculation for each He atom for that step in which the PIMD energy reaches its minimum. This geometry is basically the same as the optimized classical minimum energy shown above in panel (a) of Fig. 7. This confirms the lack of convergence towards this structure as a possible sufficiently stable configuration. But, aside from this consistency between the quantum mechanical PIMD calculation and the minimum energy geometry found classically with the EA calculation (which, on the other hand, is also seen for the cases of *N* = 6, 12 and 24) nothing else can be said regarding a special stability for this geometry.

### 4.2 SF_6_^+^

**Fig. 9:**
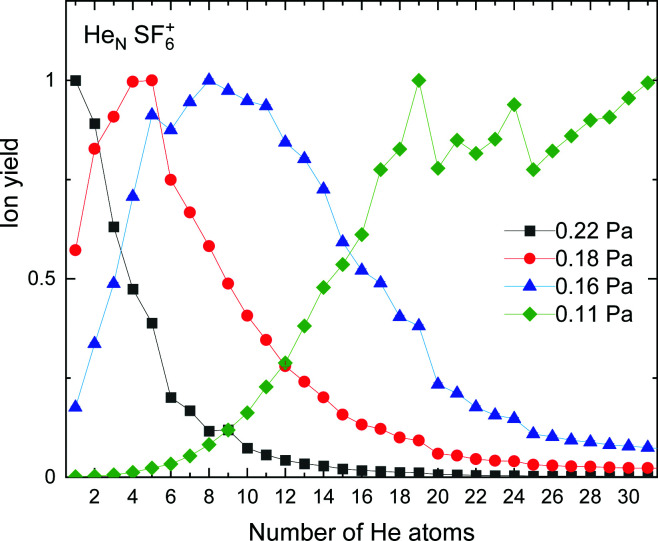
Same as Fig. 5 for He_*N*_*SF*_6_^+^ with values of gas pressures of 0.11 Pa (green diamonds), 0.16 Pa (blue triangles), 0.18 Pa (red circles) and 0.22 Pa (black squares).

The experimental ion yield for He_*N*_-SF_6_^+^, shown in Fig. 9, displays some similarities in comparison with those in the case of sulphur pentafluoride ions (see Fig. 5). The trend followed as a function of the number of He atoms also depends on the value of the gas pressure, but for some specific sizes, such as *N* = 5, 11 and 19, a significant decrease is systematically observed. Interestingly, this represents a shift to-one-He-atom smaller complexes as compared to He_*N*_–SF_5_^+^ (see Fig. 5). This result seems consistent with our previous analysis of the minimum energy configuration for He–SF_5_^+^ and He–SF_6_^+^ (shown in Fig. 4), where we found that the extra F atom occupies the position of one of the minima in the case of sulphur hexafluoride ion reserved in the case of SF_5_^+^ to a He atom. This would mean that the same stable structures are managed with one He atom less.

This suggestion is confirmed when we calculate the evaporation energies by means of the above mentioned EA to search for the location of the He atoms in their minimum energy configurations. In these classical optimizations, the extra F is located in the minimum shown in panel (b) of Fig. 4, whereas the remaining F and S atoms are fixed in their equilibrium locations.

As shown in panel (b) of Fig. 4, the classical optimization applied to one He atom yields the occupancy of the symmetrically equivalent position. Estimates made by Albertini *et al.* compared different isomers of an atom of He bound to SF_6_^+^ (see Fig. 4 from ref. 26). According to the relative energies given in that work, the most stable configuration corresponds to a geometry in which the He atom occupies a different minimum site symmetrically opposed to the extra F atom separated by a central SF_5_ core (IIa in ref. 26). However, we find that the energy for the He-SF_6_^+^ geometry shown in Fig. 4 (IIb in ref. 26) remains about 2.01 meV below that for the isomer IIa. Moreover, the relative energies of the so-called IIc isomer (with the He atom aligned in the axis F–S–F) in that reference would be about 15 meV and 13 meV with respect to the IIb and IIa isomers, respectively (instead of the 3 meV and 11 meV reported in the work by Albertini and coworkers). These results indicate the close proximity of the absolute and relative minima for this system. The slight differences in the actual energy values obtained in both studies are likely to have their origin in the geometry optimization performed in each case. On the one hand the basis set employed in ref. 26 is smaller than the one we use here, and on the other hand, the relaxation of the SF_5_^+^ core is not allowed in our approach. Extended calculations by Milan Ončák refining those values published in ref. 26 confirm our present findings.^58^

**Fig. 10:**
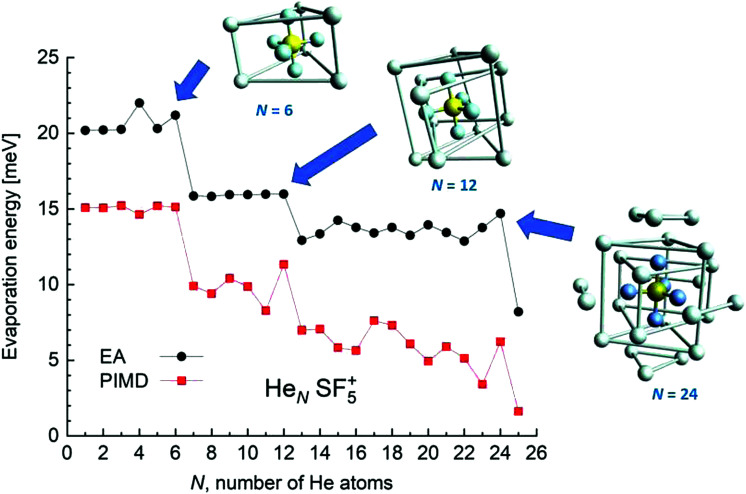
Same as Fig. 6 for the He_*N*_*SF*_6_^+^ clusters. In this case, the minimum energy structures correspond to *N*= 5, 11 and 23 droplets.

The corresponding Δ*E*_*N*_ energies as a function of *N* are shown in Fig. 10. The similarities with the sulphur pentafluoride ion are also manifested in step-like structures suggesting the onset of stable configurations once He atoms fill specific equivalent locations around the dopant. Thus, special features of the evaporation energies are then manifested at *N* = 5, 11 and 23, that is, exactly at one-He-atom-less sizes as compared with the sulphur pentafluoride ions (see Fig. 6). For those apparent magic numbers, minimum energy geometries as predicted by the EA optimization have been included in the figure. In all these three cases, the extra F atom occupies one of the six equivalent minima found for the He–SF_5_^+^ interaction, which, as we mentioned in Section 4.1, was the site reserved for one He atom.

The corresponding PIMD simulation yields evaporation energies which, once again, are in a reasonably good qualitative agreement with the classical EA predictions. Although these theoretical results allow therefore the understanding of the presence of the anomalous features at *N* = 5 and 11 observed in the experimental yields, our calculations do not explain the decrease seen at *N* = 19 for the different gas pressures.

**Fig. 11:**
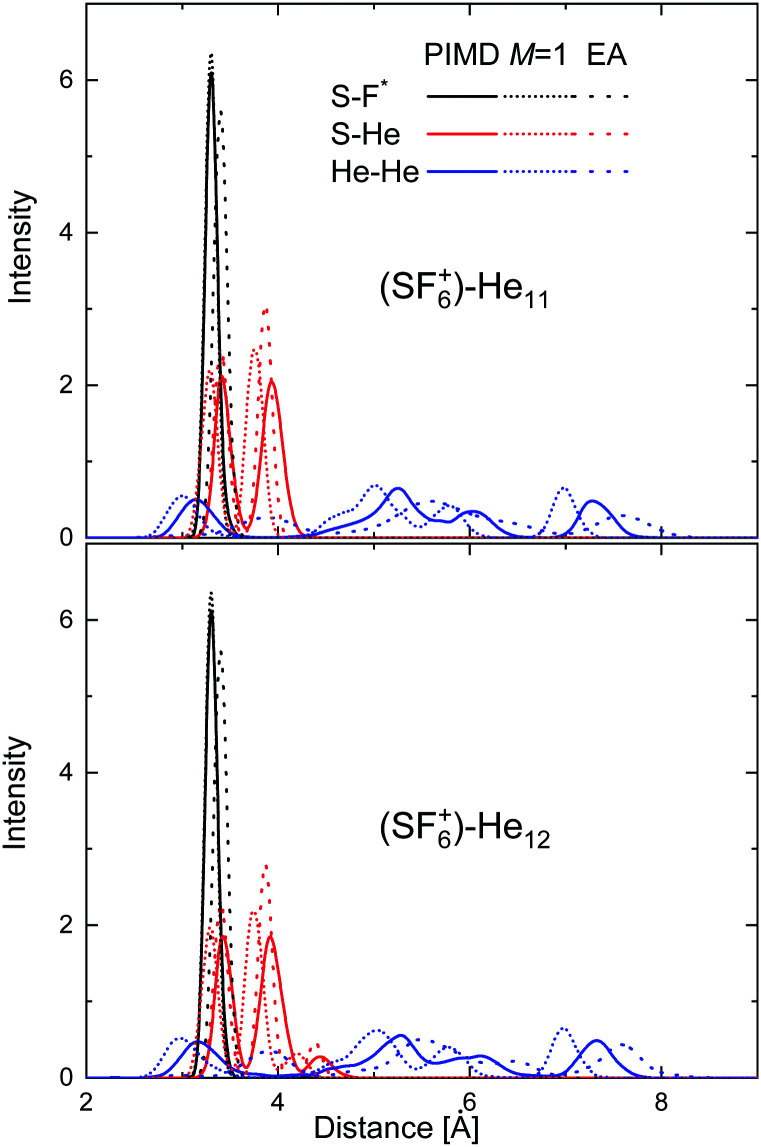
Probability densities for the S–F (black), S–He (red) and He–He (blue) distances for He_11_SF_6_^+^ (upper panel) and He_12_SF_6_^+^ clusters (bottom panel). PIMD results (solid lines) are compared with the classical approach with *M* = 1 (dashed lines) and the EA predictions (dotted lines).

In an attempt to investigate the location of both the He atoms and the extra F atom in the He_*N*_SF_6_^+^ species in more detail, radial probability densities for the different interparticle distances have been obtained. Fig. 11 shows such density functions for the S–F, S–He and He–He distances in the cases of He_11_–SF_6_^+^ and He_12_–SF_6_^+^. The figure includes the comparison between the PIMD results and those of its classical version in which the number of beads *M* = 1 (see Section 3.3). Radial distributions obtained using Gaussian functions centered at the discrete distances predicted by the EA approach are also compared, thus enabling an overall measurement of the degree of fluctuation of the quantum PIMD result. It is worth noting that in the EA global optimization, the F atom is chosen to remain fixed in the position found for the minimum of the He atoms with respect to the SF_5_ core. Fig. 11 reveals nevertheless that the quantum distribution for S–F does not differ too much with respect to the classical *M* = 1 and EA distributions. The same is seen for the S–He distance, with both the PIMD and EA distributions for He_12_-SF_6_^+^ showing that the presence of an extra He atom with respect to *N* = 11 leads to the onset of a maximum at a slightly larger distance (∼4.4 Å). As expected, more significant discrepancies are observed for the He–He distances, given the weaker interaction between helium atoms as compared to the other components. A similar comparative analysis for some other specific clusters (*N* = 5–6 and 23–24) is presented in Fig. S2–S4 of the ESI.†

## 5 Conclusions

Helium droplets surrounding the sulphur penta- and hexa-fluoride ions are investigated using experimental mass spectrometry and quantum mechanical path integral molecular dynamics calculations. The theoretical analyses of the structure and energies of the doped helium clusters He_*N*_–SF_5_^+^ and He_*N*_–SF_6_^+^ are carried out using a new intermolecular potential energy surface with *ab initio* points analytically represented *via* improved Lennard-Jones expressions whose parameters relate too fundamental physical properties of interacting partners. The characteristic features observed in the measured ion yields are explained by the existence of stable configurations of the He atoms around the impurity at specific sizes.

## Conflicts of interest

There are no conflicts to declare.

## Supplementary Material

CP-024-D1CP04725F-s001
