# Supplementary material for: Helium structures around SF5+ and SF6+: novel intermolecular potential and mass spectrometry experiments
Source: Phys Chem Chem Phys. 2021 Dec 17;24(4):2004–14. doi: 10.1039/d1cp04725f (PMC8790812; doi:10.1039/d1cp04725f)
Supplement: CP-024-D1CP04725F-s001 [file CP-024-D1CP04725F-s001.pdf]

Electronic Supplementary Material (ESI) for Physical Chemistry Chemical Physics.
This journal is © the Owner Societies 2021

Supporting Information: Helium structures around SF_5^+ and SF_6^+: A new intermolecular potential and mass spectrometry experiments

Eva Zunzunegui-Bru

Instituto de Física Fundamental, IFF-CSIC, Serrano 123, Madrid 28006, Spain

Elisabeth Gruber, Stefan Bergmeister, Miriam Meyer, Fabio Zappa and Paul Scheier

Universität Innsbruck, Institut für Ionenphysik und Angewandte Physik, Technikerstraße 25, 6020 Innsbruck, Austria

Massimiliano Bartolomei, Pablo Villarreal, and Tomás González-Lezana*

Instituto de Física Fundamental, C.S.I.C., Serrano 123, Madrid 28006, Spain

Fernando Pirani

Dipartimento di Chimica, Biologia e Biotecnologie, Universitá di Perugia, 06123 Perugia, Italy

(Dated: December 15, 2021)

*t.gonzalez.lezana@csic.es

I. IMPORTANCE OF THREE-BODY EFFECTS IN THE INTERMOLECULAR POTENTIAL

In order to estimate the role of many-body effects in the non-electrostatic components of the total interaction we have carried out additional calculations of the intermolecular potential in the He_2-SF_5^+ aggregate at the CCSD(T)/aug-cc-pVTZ level of theory as a function of the rotation angle ϕ of one He atom with respect the other one (see Figure 1). In particular, while one He atom (He (1)) is fixed at its equilibrium configuration at a distance of 3.25 Åwith respect to the S atom, the other one (He (2), also fixed at the same S-He distance) is lying on the same He-S-F plane (being F one of the axial fluorine atoms). As shown in Figure 1, a quite good agreeement is found between the total three-body estimation and that obtained as a sum of two-body contributions (those from He-He and all He-SF_5^+ interaction pairs). In particular, the two-body deviation in the minimum region ($\phi \simeq 50$ degrees) is around 1%, and it maintains a similar trend also in the repulsive region (smaller ϕ values). Therefore, these findings suggest that the present two-body model for the representation of the He_N-SF_5^+ and He_N-SF_6^+ interactions can be considered as accurate.

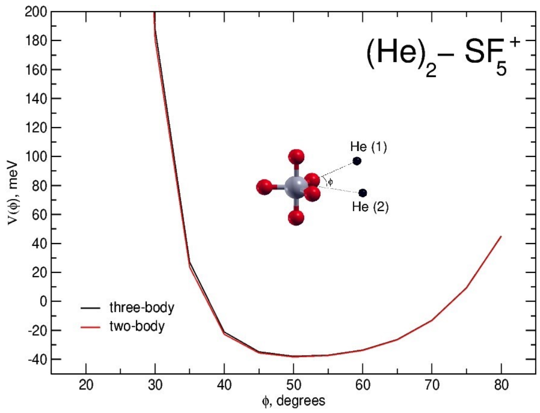

FIG. 1: Intermolecular potential for $He_2SF_5^+$ as function of the rotation angle $\phi(\mathrm{He}-\widehat{\mathrm{S}}-\mathrm{He})$ of one He atom with respect to the other one (He (1)), which is maintained fixed at its equilibrium geometry. Both He atoms are at the same S-He distance equal to 3.25 Å. Both two-body and three-body estimations are obtained at the CCSD(T)/aug-cc-pVTZ level of theory.

II. RADIAL PROBABILITY DENSITY FUNCTIONS

In order to complete the radial probability density functions shown in the manuscript for the S–F*, S–He and He–He interparticle distances , we include here the case of He$_N$-SF$_6^+$ with N =5, 6, 23 and 24. As in the Figure 11 of the manuscript, we present a comparison between distributions obtained by means of the PIMD, both with a number of beads $M = 20$ and in its classical version ($M = 1$), and EA approaches. The radial probability densities calculated with this latter global optimization algorithm has been calcualted dressing the stick discrete values of the interparticle distances with Gaussian funtions of an arbitrary width. Those selected cases correspond to those sizes which present some special feature in the evaporation energies of Fig. 10 of the manuscript. In fact the addition of an extra He atom when passing from $N = 5$ to 6 and, from $N = 23$ and 24 is reflected in the S–He densities by the onset of an extra maximum, thus indicating the location of the extra He atom in a different location with respect to the other atoms. There is no significant evolution of the S—F* probability densities when more He atoms are added to the cluster. This certainly seems to suggest that the located position of the extra fluorine atom remains unchanged with no significant effect due to the increasing number of He atoms. Despite the comparison between the classical and the PIMD distributions on the He–He interparticle distance reflects some similarities, our results confirm the quantum nature of the surrounding helium which feels a weaker interaction within the cluster than fluorine.

We also include the corresponding radial distributions for the S–He and He–He distances for the case of He$_{11}$SF$_5^+$ in Figure 6. The comparison with the results shown in Figure 11 of the manuscript for He$_{12}$SF$_6^+$ reveals that the extra F* indeed occupies the location of one of the He atoms.

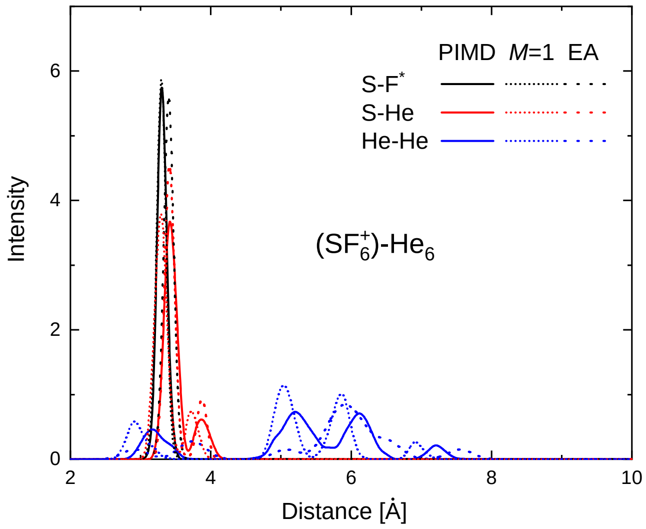

FIG. 3: Same as Figure 2 for $He_6SF_6^+$.

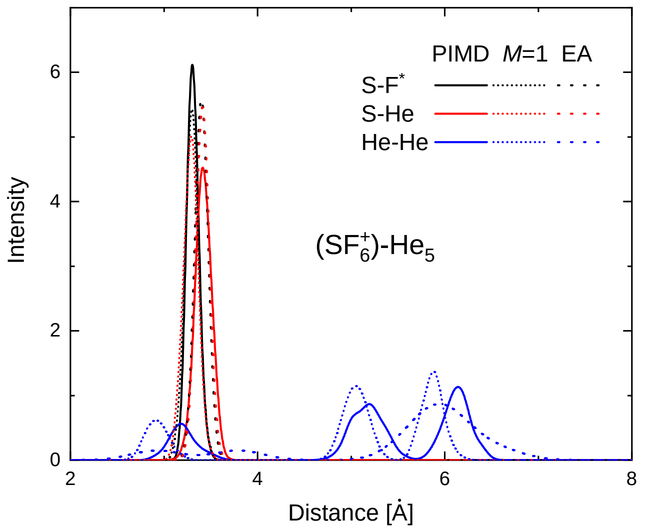

FIG. 2: Radial probability densities for the S–F* (black), S–He (red) and He–He (blue) distances for the $He_5SF_6^+$ cluster obtained by means of the PIMD with $M = 20$ (solid lines), its classical version with $M = 1$ (dashed lines) and EA (dotted lines) approaches.

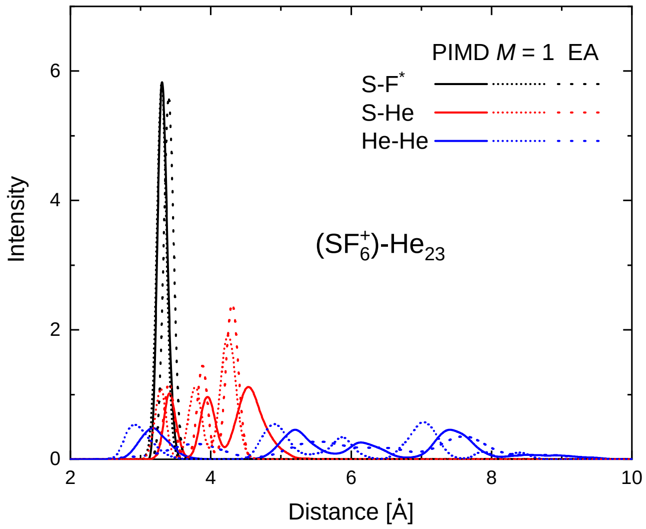

FIG. 4: Same as Figure 2 for $He_{23}SF_6^+$.

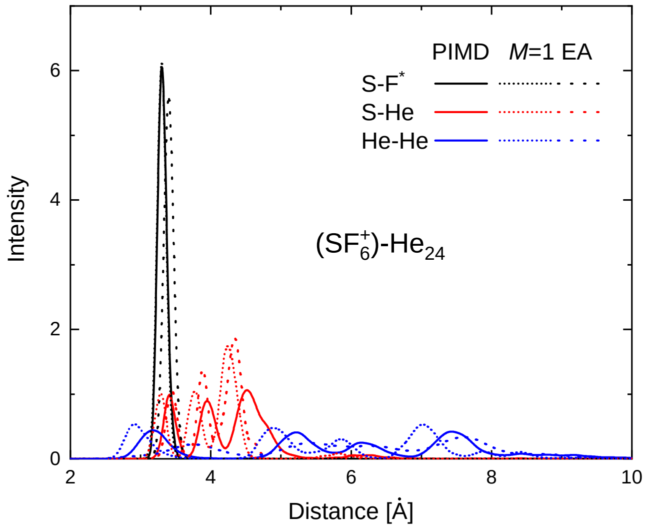

FIG. 5: Same as Figure 2 for $He_{24}SF_6^+$.

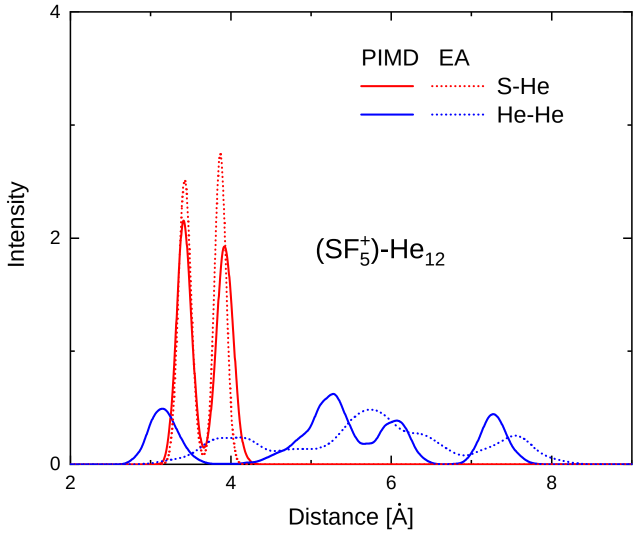

FIG. 6: Radial probability densities for the S–He (red) and He–He (blue) distances for the $He_{12}SF_5^+$ cluster obtained by means of the PIMD (solid lines) and EA (dashed lines) approaches.
